# Effect of calcium chloride on the uniformity of colouring in sushi red ginger slices by modulating the properties of starch

**DOI:** 10.1039/c8ra09025d

**Published:** 2019-01-14

**Authors:** Liping Jiao, Shuqin Xia, Xiaoming Zhang, Jianzeng Liu, Jingyang Yu, Man Zhang, Xuejiao Wang, Xiangzhen Qi

**Affiliations:** State Key Laboratory of Food Science and Technology, School of Food Science and Technology, Collaborative Innovation Center of Food Safety and Quality Control in Jiangsu Province, Jiangnan University 1800 Lihu Boulevard Wuxi Jiangsu 214122 China sqxia2013@hotmail.com sqxia@jiangnan.edu.cn +86 510 85884496; Laiwu Manhing Foods Corporation 1 Wanxing Road, Yangzhuang Town, Nonggao District Laiwu Shandong 271100 China

## Abstract

The colour of sushi red ginger slices without blanching is not uniform, which seriously affects their sensory quality. The effect of calcium chloride (CaCl_2_) pretreatment on the uniformity of colouring and the properties of ginger starch have been studied. The crystalline region of the starch in blanched ginger slices was broken, which might be beneficial for uniform colouring. The effect of CaCl_2_ pretreatment on starch properties depended on the concentration. The influence of CaCl_2_ at a concentration higher than 3.5 mol L^−1^ was more pronounced than that at a lower concentration. The uniformity of colouring was close to the effect of blanching treatment. Furthermore, the starch crystallization was destroyed, the granules were broken, and the polarized cross disappeared, which was consistent with that observed for the starch in blanched ginger slices. Therefore, it is possible to achieve a uniform colour in red ginger slices at room temperature through CaCl_2_ pretreatment.

## Abstract 2

4 mol L^−1^ CaCl_2_ change the crystallization and polarization properties of starch at room temperature, which made ginger coloring evenly.

## Graphical abstract

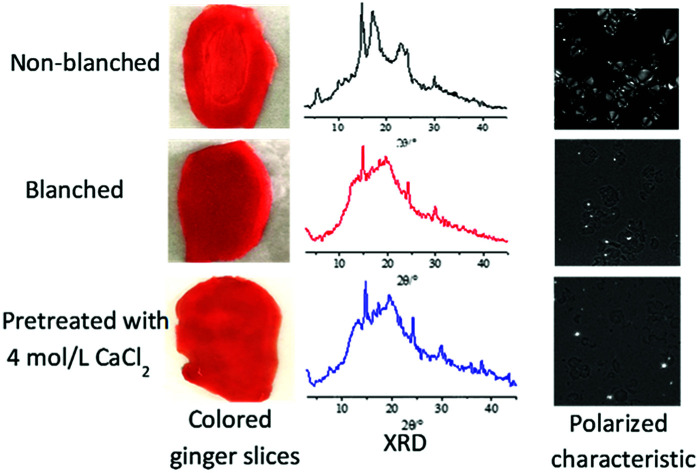

## 1. Introduction

Ginger belongs to the family Zingiberaceae, and it is a perennial grass root plant with abundant nutritional and medicinal value, such as exerting anti-oxidative, anti-tumour and hypoglycaemic effects.^1,2^ Ginger has a long history and a wide planting area in China, Vietnam and other Southeast Asian countries, and it occupies an important position in agriculture development.^3^ In many countries, fresh ginger is used to prepare dishes and as a flavouring agent in beverages.^4^ With the development of the economy and the progress of society, deep-processed products of ginger are attracting more attention, and they have broad market prospects. Among them, sushi red ginger slices is popular with consumers for its bright colour and refreshing taste and is widely exported to Europe, America and Africa.

Sushi red ginger slices is an industrial product prepared by adding colour seasoning liquid containing Allura red (E129) to desalted ginger slices after blanching at 95 °C. Allura red (2-hydroxy-1-(2-methoxy-5-methyl-4-sulphonatophe-nylazo)-naphthalene-6-sulphonate) is one of the most widely used synthetic diazo colorants.^5^ It is usually added to food products in order to achieve the desired colour and has been used in soft drinks, sweets,^6^ kimchi,^7^ a traditional Korean fermented food and others. Blanching is a traditional thermal treatment that applied to various fruits and vegetables. The main purpose of blanching is to inactivate enzymes and destroy microbial cells. However, heat treatment also causes loss of nutritional and sensorial quality attributes.^8,9^ Blanching leads to the leaching out of phytochemicals in ginger and decreases the antioxidant potential.^10^ In the processing of sushi red ginger slices, blanching ensures uniform colouring quality. However, this process consumes large quantities of energy and is a costly commercial operation.^11^ Considering energy conservation and business benefit, the industry hopes to directly add colour seasoning liquid to non-blanched ginger slices. However, we found that the problem of uneven colouring will occur during the production if the seasoning liquid contains Allura red, especially for old ginger slices. The phenomenon of uneven colouring is mainly due to the difficulty in colouring the middle part of the ginger slices and the easiness of the edge part, which seriously affects the quality of appearance of the product and, thus, affects the economic benefits.

The reason for difficulty in colouring may be related to the component of the ginger, which include water, starch, cellulose, pectin and others. The content of starch in old ginger is high, accounting for nearly 56% of the dry mass, and starch content in young ginger accounts for only about 19%. Several approaches have been made to modify starch as a potential adsorbent for dyes.^12–14^ In sewage treatment, crosslinked amphoteric starch flocculants have a strong adsorption effect and can effectively enhance flocculation and sedimentation.^15^ Starch/polyaniline nanocomposite might effectively adsorb dyes from textile effluents.^16^ Heat treatment during the blanching process is a common method of gelatinizing starch and destroying crystallization.^17^ The gelatinization onset temperature (*T*_o_) of ginger starch is 69.7 °C, peak temperature (*T*_p_) is 77.3 °C and conclusion temperature (*T*_c_) is 90.5 °C.^18^ Therefore, blanching pretreatment at 95 °C can gelatinize starch, which might facilitates the adsorption of pigment and renders the ginger colouring more even. During heat treatment, the gelatinization process mainly consists of water swelling in the non-crystalline region, gradual disappearance of the crystalline region with water entry, and cracking of the starch granules. In addition, factors such as pH, sugar, salt, lipids, and emulsifiers also have different effects on the gelatinization properties of starch.^19–22^

Salts have an obvious effect on the gelatinization of starch.^23–26^ Research has shown that different types of salt exhibited different effects on the energy required for starch gelatinization and the gelatinization temperature curve.^27^ Salting ions increased the gelatinization temperature and enthalpy of maize and waxy maize starch, and the effect of salt-soluble ions was the opposite.^28^ Furthermore, the degree of influence on the gelatinization temperature was also different. Some salts, such as NaCl and KCl, have little effect on starch gelatinization temperature.^29^ Ahmad showed that alkali metal salts (LiCl, MaCl_2_ and CaCl_2_) changed the gelatinization temperature of sago starch. When the alkali metal salt reaches a certain concentration, the starch can gelatinize at room temperature.^29^ Among the alkali metal salts, calcium chloride (CaCl_2_) exhibits the potential to be used as a food-processing aid for ginger slices considering food safety. In addition, calcium ions are often used as a firming agents in the processing of fruits and vegetables, which may have a good effect on the texture of the ginger.^30^ Because desalination is performed before the use of ginger slices, we performed the pretreatment of ginger slices with CaCl_2_ before the desalination process and removed the residual CaCl_2_ during desalination. Although many studies have been conducted to disclose the relationship between ions with starch gelatinization, clear descriptions are limited regarding the effect of ions on the property of ginger slices and starch. It is challenging to address the influence of ion concentration and environmental conditions on the property of ginger slices as a coloured matrix. These investigations further inspire us to explore a non-blanching method for ginger slices with good pigment absorption property.

To achieve energy conservation, a creative non-blanching method to evenly colour ginger slices was developed in this study. The effects of blanching on ginger starch properties were compared. The effects of CaCl_2_ concentration, processing time and temperature on the uniformity of colouring were investigated with a* value as an index. To obtain further insights on the influence of CaCl_2_ treatment on ginger slices, the dependence of starch structure on the CaCl_2_ concentration was elucidated *via* X-ray diffraction and polarization microscopy. These studies could provide guidance for improving the uniformity of colouring and quality of appearance of non-blanched red ginger slices.

## 2. Materials and methods

### 2.1 Materials

Salted ginger (*Kaempferia galanga*) slices (10% NaCl, thickness 2–3 mm) and Allura red (food grade, 98% purity) were provided by Laiwu Manhing Foods Corporation (Shandong, China). Analytical grade reagents, including sodium hydroxide, CaCl_2_ and anhydrous ethanol, were purchased from Shanghai Chemical Reagent Corporation (Shanghai, China). All aqueous solutions were prepared with deionized water (Milli-Q water).

### 2.2 Blanching treatment of ginger slices

Salted ginger slices (thickness 2–3 mm, 200 g) were immersed in a thermostatic water bath (1 : 2 w/v) at 95 °C for 5 min. After blanching, the sample was placed in deionized water for 20 min (1 : 2 w/v) to remove salts, and this process was repeated 4 times. The sushi ginger slices were prepared *via* vacuum sealing after adding the liquid material to it (2 : 1 w/v). The liquid material contains salt, edible acetic acid, citric acid, aspartame, sodium saccharin, potassium sorbate, monosodium glutamate, and 0.03% Allura red. A non-blanched sample was used as the control.

### 2.3 CaCl_2_ treatment of ginger slices

The CaCl_2_ solution was prepared with different concentration (2, 2.5, 3, 3.5, 4, 4.5 and 5 mol L^−1^). Salted ginger slices (200 g) were weighed and added to CaCl_2_ solution (1 : 2 w/v) respectively. Then the system were mixed for 10, 20, 30, 60, 90 and 120 min with a stirring paddle at 50 rpm. The processing temperature was set at 20, 30, 40 and 50 °C respectively. The ginger slices were then separated from the CaCl_2_ solution and placed in deionized water for 20 min (1 : 2 w/v) to remove salts and CaCl_2_. The desalting process was repeated 4 times. After desalting, the sushi ginger slices were prepared by vacuum sealing after adding the liquid material to it (2 : 1 w/v). The liquid material used was the same as that described above.

### 2.4 Measurement of ginger slices colouring

The colour of ginger samples were assessed using a high-precision chromometer (UltraScan Pro1166, Hunterlab Corporation, Reston, VA, USA). Before measurement, the blackboard and the whiteboard were used for zero correction. Five ginger slices were randomly chosen from each pack, and 4 points were selected on each ginger slice to determine the colour value of the ginger slices. The L* (lightness), a* (redness) and b* (yellowness) values were recorded. The average a* value and the coefficient of variation were calculated to indicate the adsorption degree of pigment on the ginger and the uniformity of ginger colouring, respectively.

To reflect the relationship between the concentration of Allura red and a* value, the a* value of the pigment solution (2, 4, 6, 8, 10 and 12 μg mL^−1^) was measured using the high-precision chromometer. The regression equation is *Y* = 2.115*X* + 8.4467 (*R*^2^ = 0.94), in which *Y* represents a* value, and *X* represents the concentration of Allura red.

### 2.5 Isolation of starch

The desalted ginger slices were macerated at a low speed in a Jiuyang blender (200 g of ginger slices : 200 g of deionized water) for 1 min. The homogenate was poured through four layers of muslin cloth, and the yellow residue that settled was washed with deionized water twice. Next, the mixture was sieved through a 100-mesh sieve to remove the residue. Ethanol was added to the filtrate to make its concentration more than 30%, followed by centrifugation at 8000 rpm for 10 min. The colourless precipitate was deproteinated using 0.01 mol L^−1^ NaOH and was washed repeatedly until the washing water was rendered neutral. The starch was collected and freeze dried (Scientz-18N, Ningbo Scientz Biotechnology Corporation, China) for 48 h. Then, the dried starch was ground with a mortar and pestle, and passed through a 200-mesh sieve to obtain starch powder which was stored in a sealed container at room temperature.^31^

### 2.6 Microstructure of starch granules

The starch and water were used to prepare starch milk at a proportion of 1 : 100 w/v. A drop of starch milk was placed onto the glass slide, and was gently shaken to evenly distribute the starch, and was covered with a glass lid. The starch samples were observed under a microscope (BX41, Olympus Corporation, Tokyo, Japan) at a 500× magnification.

### 2.7 X-ray diffraction

The X-ray diffraction patterns of the starch from ginger were analysed using a D2 PHASER X-ray powder diffractometer (Phaser, Bruker AXS Corporation, Karlsruhe, German) with Cu Kα radiation at a voltage of 300 W and a power of 2.2 kW. The scattered radiation was detected in the angular range of 3–45° (2*θ*), with a scanning speed of 2° (2*θ*) min^−1^ and a step of 0.05° (2*θ*). Starch crystallinity was analysed using the Jade5 software.

### 2.8 Data analysis

Each experiment was conducted in triplicate under the same conditions. The results were reported as the mean values ± standard deviation. Statistical analysis was performed using Origin 8.5. Analysis of variance (ANOVA) and bilateral Tukey's test (*p* < 0.05) were conducted to determine significant differences.

## 3. Results and discussion

### 3.1 Colouring difference between blanched and non-blanched ginger slices

**Fig. 1:**
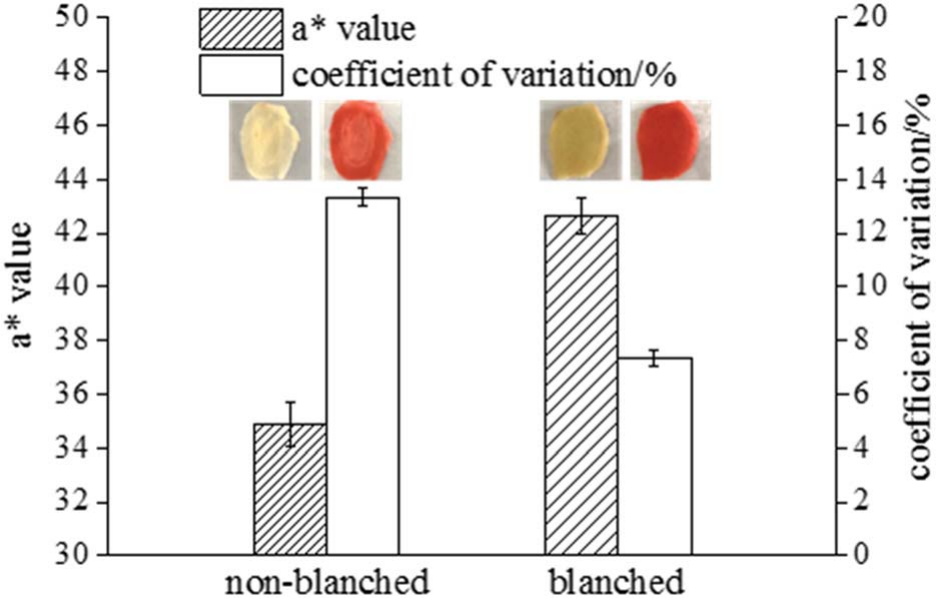
The colouring result for blanched and non-blanched ginger slices. The inserted images represent the appearances of blanched and non-blanched ginger slices before and after colouring.

To directly reflect the colouring of ginger slices, the a* value and coefficient of variation were measured. The difference between the colouring effect in the blanched and non-blanched ginger slices was obvious. The results are shown in Fig. 1.

The a* value of the blanched ginger slices increased by 22.19%, and the coefficient of variation significantly decreased by 44.87%, compared to those for the non-blanched ginger slices; this indicated that heat treatment was beneficial for pigment adsorption, and it exhibited a better colouring effect. The inserted pictures in Fig. 1 presents the appearance of blanched and non-blanched old ginger slice. The middle part of the uncoloured and untreated ginger slice was difficult to colour, and it was observed as opaque white. The content of starch in old ginger was higher than that in fresh ginger. Therefore, the whitish colour in old ginger slices might be attributed to the aggregation of more starch. When the ginger slices were coloured with Allura red, the non-blanched ginger slices exhibited poor colour uniformity because the middle part of these slices were hard to colour. The blanched ginger slices exhibited a better uniformity of colouring than the untreated ginger slices. Gelatinized starch was beneficial for the absorption of pigment by the ginger slices, and it enhanced the uniformity of colouring. The reason for this effect might be that the structure of starch was broken and more hydroxyl groups were available. The highly polar hydroxyl groups tended to form intermolecular and intramolecular hydrogen bonds.^32^ Therefore, Allura red, a hydroxyl-containing compound, could form hydrogen bonds with starch.

**Fig. 2:**
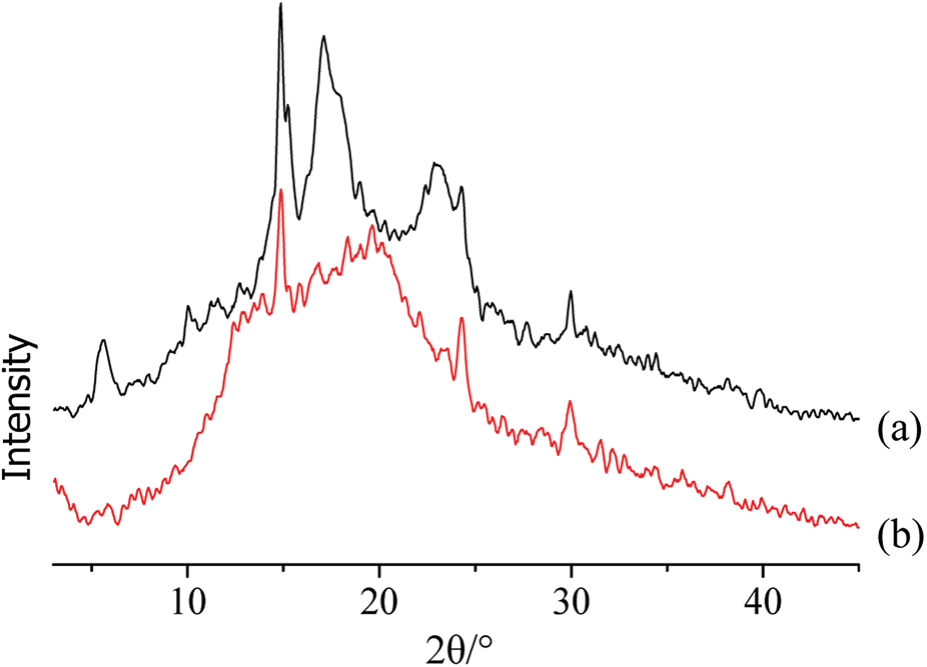
X-ray diffraction patterns of starch in non-blanched (a) and blanched (b) ginger slices.

The starch crystal characteristics of these two types of ginger slices were analysed *via* X-ray diffractometry (Fig. 2). X-ray diffraction has been used to reveal the presence and characteristics of the starch crystalline structure.^33^ The X-ray diffractogram of starch in non-blanched ginger slices showed five peaks (Fig. 2a), and the strongest diffraction peak was observed at approximately 15° at 2*θ*. A doublet between 17° and 18° and small peaks approximately 5.7° and 23° indicated that native ginger had a characteristic C-type starch.^34,35^ The crystallinity of the starch from non-blanched ginger was 19.05%; however, the crystallinity of the starch in blanched ginger slices was 0.51%. The latter no longer possessed the characteristic absorption of the C-type, which indicated that the high temperature broke the hydrogen bond and destroyed the crystalline region of starch in the ginger slices. This further proved that gelatinization of starch occurred in ginger slices after blanching.

### 3.2 Colouring difference in ginger slices treated by CaCl_2_

The blanching method was conducive to the uniform colouring of ginger slices mainly because of the gelatinization of starch and breaking of the crystalline region according to the above analysis. Existing research showed that alkali metal salts with appropriate concentration could reduce the gelatinization temperature of sago starch.^29^ Considering food safety, CaCl_2_ was selected as a food-processing aid to pretreat the ginger slices. The influence of CaCl_2_ concentration, protreatment time and temperature on the uniformity of colouring were investigated with the a* value and coefficient of variation as indicators.

**Fig. 3:**
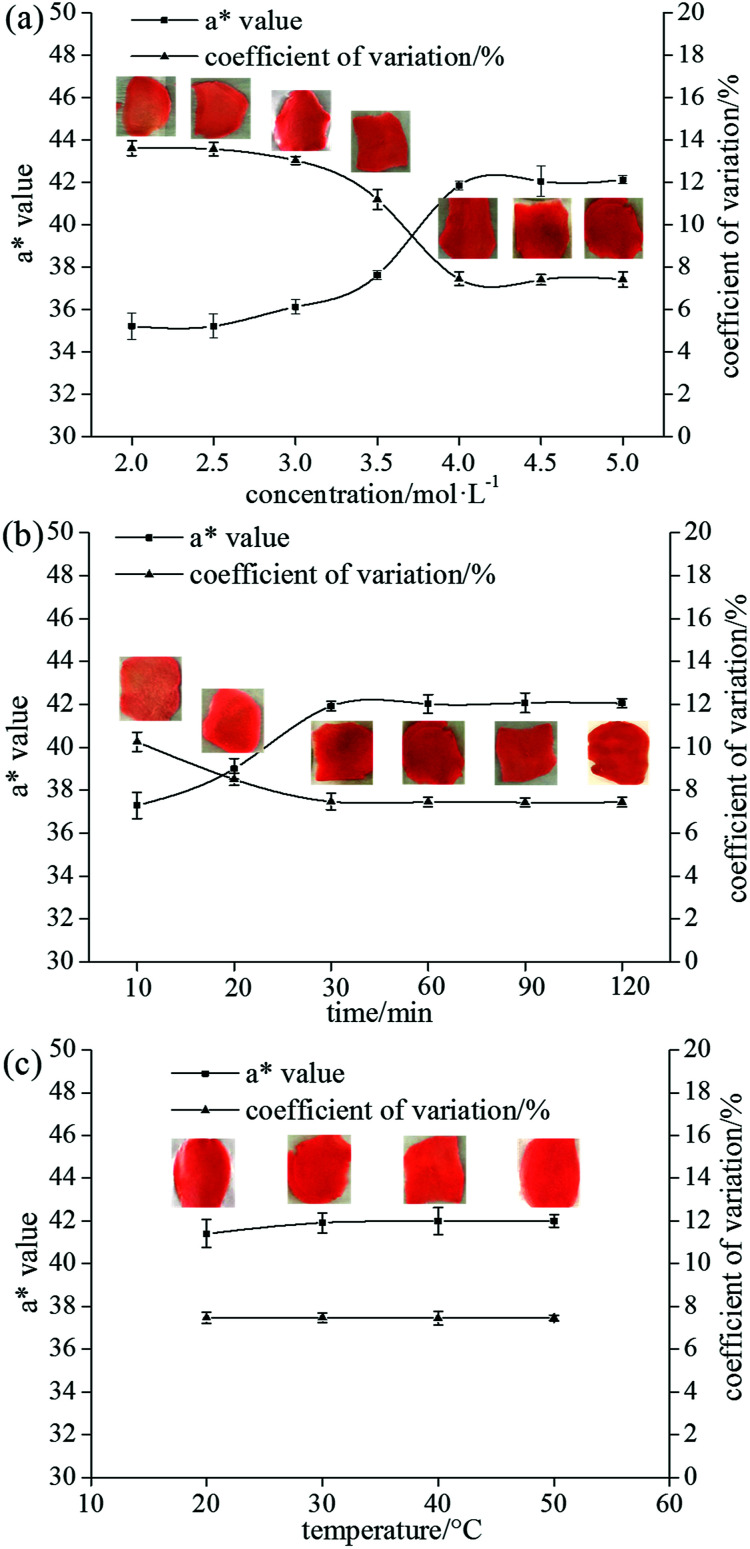
Effect of CaCl_2_ concentration (a), time for pretreatment with CaCl_2_ (b) and temperature for pretreatment with CaCl_2_ (c) on the colouring of ginger slices. (a) Salted ginger slices were soaked in CaCl_2_ solution (1 : 2 w/v) and stirred at 50 rpm and 30 °C for 30 min; (b) the salted ginger slices were soaked in 4 mol L^−1^ CaCl_2_ solution (1 : 2 w/v) and stirred at 50 rpm and 30 °C for different time periods; (c) the salted ginger slices were soaked in 4 mol L^−1^ CaCl_2_ solution (1 : 2 w/v) and stirred at 50 rpm and different temperatures for 30 min. The inserted images represent ginger slices coloured using Allura red.

Fig. 3a shows the dependence of the ginger-slice colouring on the concentration of CaCl_2_. The concentration of CaCl_2_ exhibited an important influence on the colouring of the ginger. When the concentration of CaCl_2_ was less than 3.5 mol L^−1^, the a* value and its coefficient of variation were similar to those of the untreated ginger slices, and the colour uniformity of the ginger slices was not improved. When the concentration of CaCl_2_ was 3.5 mol L^−1^, the a* value increased by 7.83% compared to non-blanched ginger slices, and the coefficient of variation decreased by 16.18%. The uniformity of colouring of the ginger slices improved slightly, but the effect was not obvious. The a* value increased by approximately 20%, and the coefficient of variation reduced by nearly 45% when the concentration was high (≥4 mol L^−1^), which was similar to that of the blanched ginger slices. Moreover, the colouring effect of the ginger slices improved significantly. Therefore, 4 mol L^−1^ was determined to be the best concentration. However, studies had shown that 3.5 mol L^−1^ CaCl_2_ could gelatinize starch at room temperature.^29^ It is possible that the concentration was slightly lower because the study was conducted in a pure starch system and the contact area was wider.

The influence of pretreatment time on the colouring of the ginger slices is shown in Fig. 3b. Pretreatment time slightly affected the colour. The a* value increased, and the coefficient of variation decreased with the extension of the processing time (10–30 min). The colouring of the ginger slices following 30 min of treatment was similar to that of the blanched ginger slices, and the colouring tended to be stable with longer processing times. Therefore, 30 min was determined to be the best processing time.

The influence of pretreatment temperature on the colouring of the ginger slices is shown in Fig. 3c. Pretreatment temperature had little effect on the uniformity of ginger colouring (Fig. 3c). The a* value and its coefficient of variation showed a steady trend in the range of 20–50 °C, indicating that the effect of temperature was not obvious and that the treatment could be performed at room temperature.

The experimental results showed that the pretreatment time and temperature had little effect on the colouring of ginger slices. When the pretreatment time was less than 30 min, the colouring improved with increasing time. However, the effect of time was not obvious when the time was more than 30 min. This might be because CaCl_2_ could not be in full contact with starch in a short period of time. A certain concentration of salt solution could change the starch gelatinization temperature.^28,36^ CaCl_2_ at 4 mol L^−1^ had already lowered the starch gelatinization temperature to room temperature; therefore, changing the temperature had no effect on the result.

### 3.3 Effect of CaCl_2_ treatment on starch properties of ginger slices

Blanched sushi ginger slices were more likely to exhibit even colouring because the high temperature of 95 °C changed the properties of the starch in the ginger slices. The effect of different concentrations of CaCl_2_ on the colouring of sushi ginger was different. A high concentration of CaCl_2_ (≥4 mol L^−1^) produced a colouring effect in the ginger close to that obtained with the blanching treatment; therefore, this concentration might also transform starch properties. The crystallization characteristics, starch particle shape and other properties were compared by separating the starch from ginger slices that were treated with different concentrations of CaCl_2_.

#### 3.3.1 X-ray crystallography

**Fig. 4:**
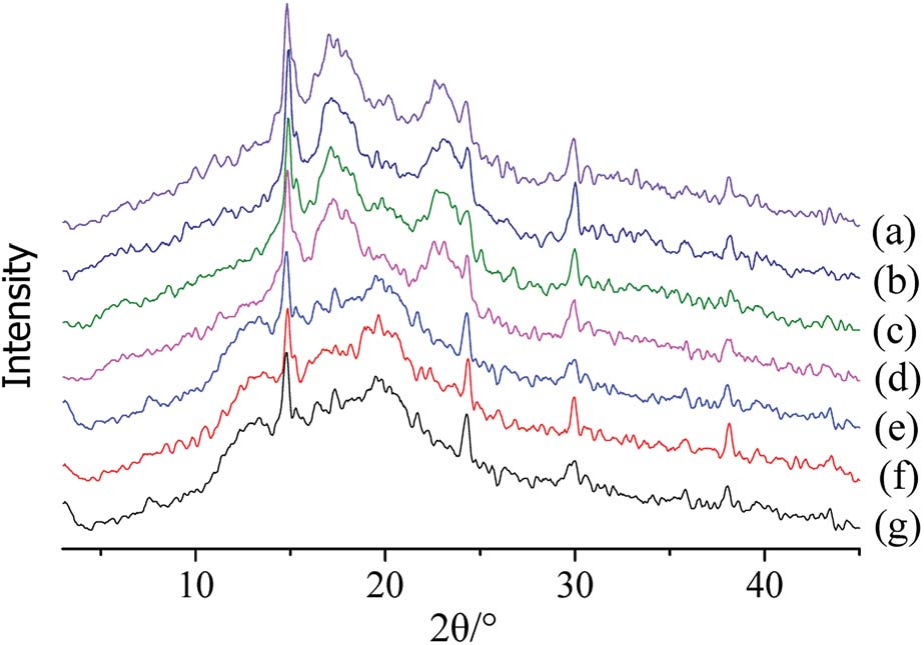
X-ray diffraction patterns of starch after pretreatment of ginger slices with different concentrations of CaCl_2_. (a) 2 mol L^−1^ CaCl_2_; (b) 2.5 mol L^−1^ CaCl_2_; (c) 3 mol L^−1^ CaCl_2_; (d) 3.5 mol L^−1^ CaCl_2_; (e) 4 mol L^−1^ CaCl_2_; (f) 4.5 mol L^−1^ CaCl_2_; (g) 5 mol L^−1^ CaCl_2_.

As shown in Fig. 4, when the concentration of CaCl_2_ was less than 3.5 mol L^−1^, the crystalline structure of the starch was consistent with that from the non-blanched ginger, and the characteristic absorption peaks were obvious. The strongest diffraction peak was observed at approximately 15° at 2*θ*; a doublet between 17° and 18° and small peaks at approximately 5.7° and 23° were observed. The crystallinity was approximately 20% (19.46% for starch treated with 2 mol L^−1^ CaCl_2_, 20.11% for starch treated with 2.5 mol L^−1^ CaCl_2_, and 20.55% for starch treated with 3 mol L^−1^ CaCl_2_), which was also similar to the crystallinity for the non-blanched ginger starch. The characteristic absorption peaks of starch slightly decreased when CaCl_2_ was 3.5 mol L^−1^. The intensity of the peaks in the diffractogram could reflect the strength of the starch double-helix structure, and decrease in the reflections implied change in the structure.^37,38^ Therefore, this result indicated that the double-helix structure of starch was weakened, and the starch crystalline structure was slightly damaged; a crystallinity of 15.13% could also prove this inference. When the concentration of CaCl_2_ was ≥4 mol L^−1^, the crystallinity of starch was decreased by more than 95%. The crystallinity for starch treated with 4 mol L^−1^ CaCl_2_, 4.5 mol L^−1^ CaCl_2_, and 5 mol L^−1^ CaCl_2_ was 0.93%, 0.77%, and 0.33%, respectively. The X-ray diffractogram for the starch was similar to that of starch from blanched ginger, and the decrease in crystallinity might be due to the double-helical movement during treatment, which might have disrupted starch crystallites.^39^ Therefore, low concentrations of CaCl_2_ (<3 mol L^−1^) did not change the crystal form and the crystallinity of starch was similar to that of the non-blanched ginger slices. CaCl_2_ at 3.5 mol L^−1^ reduced the crystallinity of starch by 20.58%. However, a high concentration of CaCl_2_ (≥4 mol L^−1^) broke the crystalline structure and achieved the same effect as in the starch from blanched ginger slices.

#### 3.3.2 Particle morphology and polarized characteristic

**Fig. 5:**
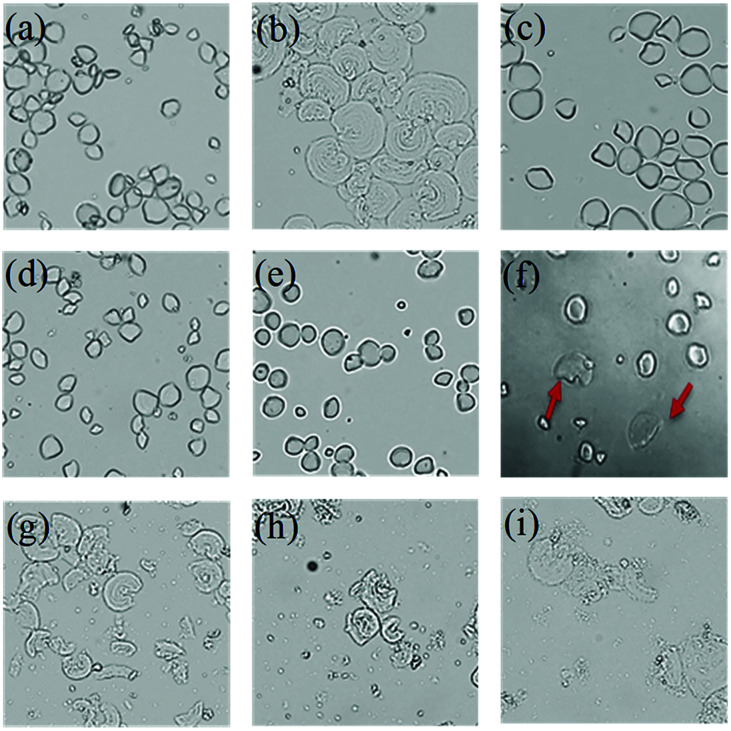
Morphological characteristics of starch granules (×500). (a) Starch from non-blanched ginger slices; (b) starch from blanched ginger slices; (c) starch from ginger slices treated with 2 mol L^−1^ CaCl_2_; (d) 2.5 mol L^−1^ CaCl_2_; (e) 3 mol L^−1^ CaCl_2_; (f) 3.5 mol L^−1^ CaCl_2_; (g) 4 mol L^−1^ CaCl_2_; (h) 4.5 mol L^−1^ CaCl_2_; (i) 5 mol L^−1^ CaCl_2_. The red arrows indicate destroyed starch granules.

CaCl_2_ pretreatment could influence the properties of starch, and the particle morphology of starch also changed accordingly. The morphology of starch from ginger slices was observed using a light microscope (Fig. 5).

The starch structure is different for different sources.^40^ The starch granule shape for non-blanched ginger slices appeared to be oval or irregular polygonal (Fig. 5a). The starch granule shape for blanched ginger slices changed and exhibited an increase in size (Fig. 5b). In this process, the granules swelled and broke, and the structure was destroyed. Different concentrations of CaCl_2_ had different effects on starch morphology. When the concentration of CaCl_2_ was less than 3.5 mol L^−1^, there was no obvious change in the morphology of the starch granules, which remained oval or polygonal (Fig. 5c–e). When CaCl_2_ was 3.5 mol L^−1^, there was no effect on most of the starch granules (Fig. 5f). However, it could be seen that edges became blurred for a small amount of starch granules, and the shape of the granules changed. When the concentration of CaCl_2_ was more than 4 mol L^−1^ (Fig. 5g–i), almost all starch granules were destroyed, which was consistent with the result obtained for the starch from blanched ginger.

**Fig. 6:**
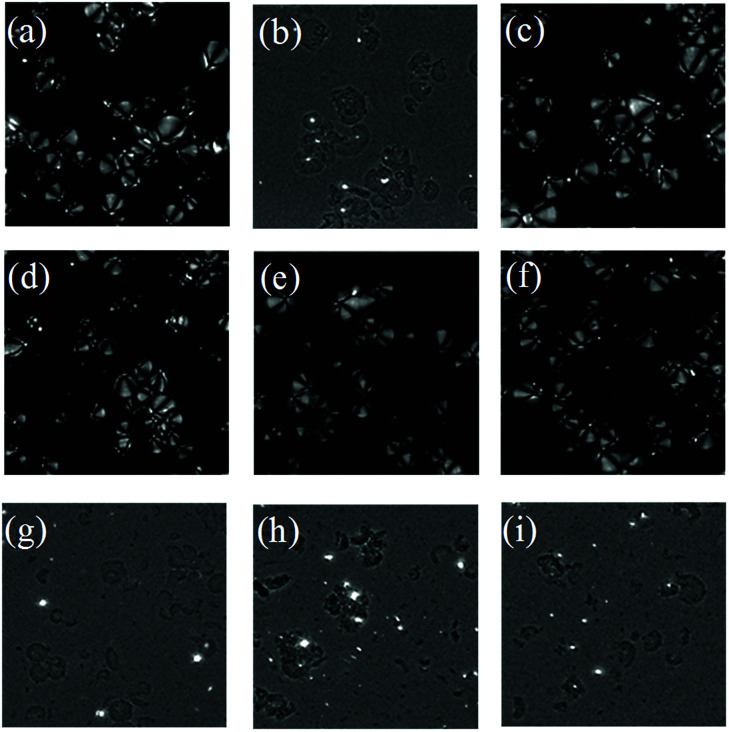
Polarization characteristics of starch granules (×500). (a) Starch from non-blanched ginger slices; (b) starch from blanched ginger slices; (c) starch from ginger slices treated with 2 mol L^−1^ CaCl_2_; (d) 2.5 mol L^−1^ CaCl_2_; (e) 3 mol L^−1^ CaCl_2_; (f) 3.5 mol L^−1^ CaCl_2_; (g) 4 mol L^−1^ CaCl_2_; (h) 4.5 mol L^−1^ CaCl_2_; (i) 5 mol L^−1^ CaCl_2_.

The starch granules were spherical crystals that were birefringent under polarized light microscopy. There were different structural regions within the starch, divided into crystalline regions and amorphous regions. The arrangement of starch chains in the crystalline regions was regular and that in the amorphous regions was disorganized. The starch chains were anisotropic, rendering the starch grains polarized.^41^ Once the crystalline region of the starch granules was destroyed, the anisotropy vanished, and the polarization disappeared; therefore, the starch structure and crystallographic information can be identified through change in the polarization of the starch granules. The polarization of starch was observed using a polarized microscope (Fig. 6).

The position and degree of visibility of the polarizing cross were related to the type of native starch granules. The starch granules from non-blanched ginger slices exhibited an obvious polarized cross, and the umbilical point was located at one end of the granules (Fig. 6a). After the blanching treatment of ginger slices, the polarized cross of the starch particles disappeared, and only broken starch granules could be seen (Fig. 6b). Different concentrations of CaCl_2_ induces different effects on the polarization of starch. When the concentration of CaCl_2_ was lower than 3.5 mol L^−1^ (Fig. 6c–e), there was still a clear polarization. Treatment with 3.5 mol L^−1^ CaCl_2_ influenced a small amount of starch granules, according to a previous analysis; however, it did not affect the polarization (Fig. 6f). Starch granules without crystalline regions lost their polarized cross under the polarized microscope.^42^ 3.5 mol L^−1^ CaCl_2_ did less damage to the crystalline region and the starch maintained a relatively high degree of crystallinity; therefore, it still retained the polarized cross. When the concentration of CaCl_2_ was higher than 4 mol L^−1^, the polarization cross completely disappeared (Fig. 6g–i), indicating that the crystalline region of the starch was destroyed at this time, which was consistent with the result for the starch from blanched ginger.

Starch granules from blanched ginger slices were broken under light microscope. Research had shown that microwave-treated starch granules that were heated to a final temperature of 95 °C appeared completely ruptured.^41^ Furthermore, through microscopic observation, Jangchud found that starch granules from blanched flours were broken and concluded that the starch was gelatinized.^17^ Therefore, the gelatinization of the starch from blanched ginger slices might be due to the blanching treatment. Additionally, the polarized cross of the starch particles disappeared under polarized microscope. This could also explain that the blanching treatment of ginger slices destroyed the starch crystal region, broke the hydrogen bonds and caused the starch to gelatinize. A high concentration of CaCl_2_ (more than 4 mol L^−1^) could also break the starch granules and induce the polarized cross to disappear, which was consistent with the result for blanched ginger starch. This result showed that at room temperature, a high concentration of CaCl_2_ (more than 4 mol L^−1^) could lead to hydrogen-bond breaking and the gelatinization of ginger starch.

## 4. Conclusions

The uneven colour of ginger slices without blanching pretreatment is related to the characteristics of starch. The crystalline region of the starch is broken in blanched ginger slices. The properties of starch were evaluated in ginger slices treated with CaCl_2_. The experiments showed that different concentrations of CaCl_2_ had different effects on the starch crystallization structure, morphology, and polarization cross in ginger slices. Pretreatment with 4 mol L^−1^ CaCl_2_ almost completely destroyed the crystallization structure of the starch in ginger slices, broke the starch granules and led to the disappearance of the polarized light cross; this effect was close to that observed with blanching treatment. A high concentration of CaCl_2_ might be used in the pretreatment of ginger slices instead of the traditional blanching treatment. It could also cause the starch to gelatinize at room temperature, and the sushi red ginger slices exhibited even colouring and good sensory qualities.

## Conflicts of interest

The authors have declared no conflict of interest.

## Supplementary Material
